# Distinct Roles of Velvet Complex in the Development, Stress Tolerance, and Secondary Metabolism in *Pestalotiopsis microspora*, a Taxol Producer

**DOI:** 10.3390/genes9030164

**Published:** 2018-03-14

**Authors:** Oren Akhberdi, Qian Zhang, Dan Wang, Haichuan Wang, Xiaoran Hao, Yanjie Liu, Dongsheng Wei, Xudong Zhu

**Affiliations:** 1 State Key Program of Microbiology and Department of Microbiology, College of Life Sciences, Nankai University, Tianjin 300071, China; oren79@163.com (O.A.); cheercheung1991@gmail.com (Q.Z.); nkwangdan@163.com (D.W.); wanghaichuansn@163.com (H.W.); weidongsheng@nankai.edu.cn (D.W.); 2 Beijing Key Laboratory of Genetic Engineering Drug and Biotechnology, Institute of Biochemistry and Biotechnology, College of Life Sciences, Beijing Normal University, Beijing 100875, China; hxrr_563@163.com (X.H.); maynur79@163.com (Y.L.)

**Keywords:** LaeA, VeA, VelB, secondary metabolism, pestalotiollide B, conidiation, *Pestalotiopsis microspora*

## Abstract

The velvet family proteins have been shown to play critical roles in fungal secondary metabolism and development. However, variations of the roles have been observed in different fungi. We report here the observation on the role of three velvet complex components VeA, VelB, and LaeA in *Pestalotiopsis microspora*, a formerly reported taxol-producing fungus. Deletion of individual members led to the retardation of vegetative growth and sporulation and pigmentation, suggesting critical roles in these processes. The mutant strain *△velB* appeared hypersensitive to osmotic stress and the dye Congo red, whereas *△veA* and *△laeA* were little affected by the pressures, suggesting only *velB* was required for the integrity of the cell wall. Importantly, we found that the genes played distinct roles in the biosynthesis of secondary metabolites in *P. microspora*. For instance, the production of pestalotiollide B, a previously characterized polyketide, required *velB* and *laeA*. In contrast, the *veA* gene appeared to inhibit the pestalotiollide B (PB) role in its biosynthesis. This study suggests that the three components of the velvet complex are important global regulators, but with distinct roles in hyphal growth, asexual production, and secondary metabolism in *P. microspora*. This work provides information for further understanding the biosynthesis of secondary metabolism in the fungus.

## 1. Introduction

*Pestalotiopsis* fungi, known as the —*E. coli*— of rain forests, are widely distributed as either common endophytes or pathogens in tropical and temperate ecosystems [1,2]. The fungi have received considerable attention in the past decades, not only for its pathogenic role in crops, but more importantly, for its capacity to produce a myriad of diverse metabolites [3,4,5,6,7,8]. In the genus, *P. microspora* is the most encountered species and serves as a model for the study of the biology and ecology of fungi [3,7]. In particular, *P*. *microspora* has been reported to produce the anti-tumor drug taxol (paclitaxel) by several laboratories all over the world [2,3,7].

The velvet family proteins are featured within the conserved velvet domain, which to date has only been found in filamentous fungi and holds important roles in protein-protein interaction and DNA binding [9,10]. The velvet family consists of four members: VeA, VelB, VelC, and VosA [10]. In several fungi, the velvet proteins are characterized as a global regulator of gene expression in many biological processes of growth, development, and secondary metabolism [11,12,13,14,15,16]. For instance, VeA regulates the expression of several genes in the synthesis of the mycotoxin sterigmatocystin and the antibiotic penicillin in *Aspergillus nidulans* [13,14]. Although VeA in this fungus represses the transcription of the isopenicillin synthetase gene *ipnA*, an enzyme required for penicillin biosynthesis, it is necessary for the expression of *acvA*, the key gene in the first step of penicillin biosynthesis [13]. In *Fusarium fujikuroi*, the velvet protein FfVel1 (VeA counterpart) plays a positive regulatory role for the biosynthesis of gibberellins, fumonisins, and fusarin C, but has an inhibitory part in the production of bikaverin. Ffvel1 also affects conidiation and virulence in this plant pathogenic fungus. The velvet protein FfVel2 (VelB) has similar functions in conidiation, secondary metabolism, and virulence as FfVel1 [15]. In *Fusarium oxysporum*, the deletion of *veA* and *velB* equivalents caused a derepression of conidiation and alterations in the morphology and size of microconidia. Furthermore, the VeA and LaeA were required for full virulence of the fungus on tomato plants [16]. One distinctive action of the velvet complex in the regulation of target genes is to respond to light change. In the dark, activated VeA takes the bound VelB into the nucleus and forms the velvet complex with LaeA, and subsequently performs a regulatory function [17]. The global regulator LaeA has been predicted to encode a methyltransferase [18,19]. In *Aspergillus* spp., deletion of *laeA* causes a marked reduction in the production of several secondary metabolites, e.g., gliotoxin, sterigmatocystin, aflatoxin, and endocrocin [18,20,21,22]. LaeA is one of the major regulators in asexual and sexual development in *A. nidulans* [19].

The endophytic fungus *P. microspora*, NK17, was previously isolated by our laboratory as a producer of taxol-like molecules [23]. Most recently, a polyketide derivative, pestalotiollide B, that is structurally analogous to the inhibitor of cholesterol ester transfer protein (CETP), dibenzodioxocinones, has also been isolated from this strain [24]. As one of our efforts to understand the biosynthetic pathways of this fungus, and to find out the role of light in the regulation of secondary metabolism in endophytes, we conducted analysis on the velvet complex members VeA, VelB, and LaeA by creating loss-of-function mutants for the genes. We present the results below.

## 2. Materials and Methods

### 2.1. Fungal and Bacterial Strains, Culture Conditions

*P. microspora* NK17 was previously isolated by our laboratory as a taxol-producing strain [23]. The uracil auxotrophic strain, NK17-*△ura3*, was created by us [25] and used as the recipient strain in the construction of the deletion mutants.

Unless otherwise specified, fungal strains were grown or maintained on potato lactose agar (PLA, 20% peeled and sliced potato, 1.0% lactose and 2.0% agar, natural pH), PLB (PLA without agar), or minimal medium (MM) [26]. All fungal strains were grown at 28 °C under normal illumination of the indoor light condition and the liquid culture was shaken at 180 rpm.

The plasmids pOSCAR and pA-Ura3-OSCAR were used for the construction of the transformation vector for gene deletion. pOSCAR was amplified in the *E. coli* strain DB3.1, and pA-Ura3-OSCAR or other manipulations were propagated in the *E. coli* DH5α strain. The *Agrobacterium tumefaciens* strain LBA4404 was used for ATMT (*A. tumefaciens*-mediated transformation) of NK17. The bacterial strains were grown in Luria–Bertani (L-B) medium and supplemented with appropriate antibiotics when needed at 28 °C for *A. tumefaciens* or 37 °C for *E. coli*, with shaking at 180 rpm. Inducing medium (IM) containing acetosyringone (AS) 40 mg·L^−1^ for induction and yeast nitrogen base (YNB) medium were used in the transformation of NK17. The mutant spores were harvested from the PLB medium.

### 2.2. Construction of Deletion Vector

Deletion vectors were constructed by the protocol of OSCAR described previously [27]. The plasmid pOSCAR provided the spectinomycin resistant gene (spect) for selection and the plasmid pA-Ura3-OSCAR [25] provided the marker gene *ura3* for uracil auxotrophic deficiency in NK17-*△ura3*.

The primers used in this study are shown in Table 1. The pair, VeA-up-s/VeA-up-as, containing attB2r and attB1r, respectively, were designed to amplify a 744-bp 5′-flanking fragment of *veA* gene. The 314-bp sequence of a 3′-flanking fragment was amplified from the NK17 genome using the primers VeA-down-s/VeA-down-as, containing the attB4 and attB3 sequences, respectively. The two polymerase chain reaction (PCR) products were then gel purified before cloning by the AxyPreP DNA Gel Extraction Kit (Axygen, Corning, NY, USA).

The deletion construct was set up using the BP Clonase II enzyme (Invitrogen, Carlsbad, CA, USA). Then, the reaction mixture was transformed into *Escherichia coli* DH5α and plated on lysogeny broth (LB) plate with 100 μg/mL spectinomycin incubated at 37 °C. The spectinomycin resistance colonies were selected and the correct deletion construct pOSCAR-VeA was verified with PCR amplifications. Through a similar procedure, the deletion plasmids pOSCAR-VelB or pOSCAR-LaeA were constructed.

### 2.3. Disruption and Restoration of veA, velB and laeA by Agrobacterium Tumefaciens-Mediated Genetic Transformation

The vector pOSCAR-VeA for *veA* disruption was introduced into *A. tumefaciens* LBA4404 by a protocol described previously [25,28]. 10^8^ CFU (colony-forming units) of LBA4404 cells containing the deletion plasmid and a number of 10^7^ conidia of the recipient fungal strain NK17-*△ura3* were co-cultured on a nitrocellulose filter, which was placed on an inducing medium (IM) plate supplemented with 50 mg·L^−1^ uracil (for the germination of spores) and 40 mg·L^−1^ acetosyringone (Sigma-Aldrich, St. Louis, MO, USA) at 28 °C. After two days of induction, the nitrocellulose filter was transferred to the yeast nitrogen base (YNB) plate containing cefotaxime (100 mg·L^−1^) for two days at 28 °C for the sporulation of fungal transformants. The individual fungal transformants were obtained by single-spore isolation on PLA containing 200 μg/mL cefotaxime (for the inhibition of the bacteria). The deletion strains *△velB* and *△laeA*, were created using the same procedure.

The complement vector carrying the wild-type copy of *veA* was constructed by the BP Clonase reaction including 40 ng *veA* allele of NK17, 60 ng pOSCAR4.1, and 1 mL BP Clonase II enzyme mix, adding double distilled water to 5 mL. The *veA* allele was amplified from the genome of NK17 using primers VeA-up-s/VeA-up-as containing the corresponding attB sequences (Table 1). The complement vector was transformed into the spores of the *△veA* strain with ATMT for the restoration test [28]. The gene *velB* or *laeA* was restored by the same method.

### 2.4. Characterization of Deletion and Restoration Strains

PCR amplification and Southern blotting were conducted for the characterization of deletion and restoration in the transformants. Genomic DNA was extracted from fresh mycelium grown in 100 mL PLB for 3–4 days [29]. Two pair of primers, VeA(s)/Ura3(as) and VeA(as)/Ura3 (s), were used for PCR analysis of the *△veA* strain.

For Southern blotting, double-digested genomic DNA by *Xba* I and *EcoR* V of the *△veA* mutant, *△veA*-C and the NK17-*△ura3* strain (as control) were separated by electrophoresis on 0.8% agarose gel and transferred onto a Magma Probe Nylon Transfer Membrane-N^+^ (Osmonics, Minnetonka, MN, USA). DNA labeling, hybridization, and detection procedures were carried out by following the protocols of the DIG High Prime DNA Labeling and Detection Starter Kit II (Roche China, Shanghai, China). The membrane was hybridized with a 1.7 kb probe generated by PCR amplification with primer pair VeA-up-s/VeA-down-as. For confirmation of the deletion of *velB* and *laeA*, PCR, and Southern blotting were likewise carried out.

### 2.5. RNA Preparation, Reverse-Transcription Polymerase Chain Reaction , and Quantitative Real-Time Polymerase Chain Reaction. 

Total RNA was isolated from lyophilized mycelia using the TRIzol Kit (Invitrogen, Carlsbad, CA, USA). To remove contaminating genomic DNA, the RNA samples were treated with RNase-free DNase (Takara, Beijing, China). Reverse-transcription PCR (RT-PCR) and quantitative real-time PCR (qRT-PCR) were performed as previously described [30].

To examine gene expression, messenger RNA (mRNA) was amplified by RT-PCR. Total RNA was isolated from fresh mycelia grown in PLB at 28 °C for 2, 3, 4, 6, and 8 days under normal illumination of the indoor light condition, respectively. Double stranded complementary DNA (cDNA) was synthesized using a M-MLV (Moloney Murine Leukemia Virus) RTase cDNA Synthesis Kit (Takara), followed by RT-PCR for 29 cycles as determined beforehand. The mRNA of the actin encoding gene *act1* served as the control. qRT-PCR was performed on a Mastercycler PCR machine (Eppendorf, Hamburg, Germany). Each reaction (20 mL) was carried out with the SYBR Green I PCR Master Mix (Roche China, Shanghai, China). Reactions were set up in duplicate. Controls without the addition of template were included for each primer pair. PCR cycling parameters were: denaturation at 94 °C for 10 min, followed by 40 cycles of denaturation at 94 °C for 15 s, annealing at 59 °C for 30 s, and extension at 72 °C for 32 s. The qRT-PCR data were analyzed using the 2^−^^△△*C*t^ relative quantification method. The mRNA of the actin encoding gene served as the internal reference. The amplification efficiencies of the target and reference genes were compared at different template concentrations.

### 2.6. Mycelial Growth and Sporulation Assessment

To characterize the fungal phenotype, 5 × 10^4^ of spores from each strain were inoculated on PLA at 28 °C. For sporulation assessment, a plug with a diameter of 10 mm was punched from the 8-day PLA culture and transferred to a centrifuge tube with 10 mL 0.8% Tween 80, and then vortexed briefly. The spores were diluted and counted with the hemocytometer. The spore production in a unit area (mm^2^) was determined. Mycelial growth was also determined by measuring two perpendicular diameters of the growing colonies on minimal medium (MM) plate daily over seven days. Experiments were carried out in triplicate Petri dishes.

### 2.7. Profiling of Secondary Metabolites by High-Performance Liquid Chromatography.

General profiling for observing the secondary metabolites produced by the fungal strains was conducted with high performance liquid chromatography (HPLC) analysis. To analyze the secondary metabolites, equal numbers of conidia were cultured in 200 mL PLB at 28 °C with shaking at 180 rpm for seven days in triplicate. The mycelium was separated from the medium by vacuum filtration. The liquid phase was extracted with an equal volume of dichloromethane. The organic phase was taken for evaporation to 1 mL at 45 °C. The final condensed suspension from the same strain (three flasks) was put together and passed through a Millipore filter (0.45 μm) for HPLC profiling. HPLC analysis was conducted at room temperature on a Kromasil C18 ODS column of 4.6 × 250 mm (AKZO Nobel, Gland, Switzerland) with a mobile phase that consisted of methanol/H_2_O (70:30, *v*/*v*; pH 7.0) at a flow rate of 1 mL/min on an Angilent 1100 system (Angilent Technologies, Santa Clara, CA, USA), and 20 μL of each sample was loaded. The peaks were detected with an ultraviolet detector at 227 nm. The yield of pestalotiollide B (PB) was quantified using a standard curve that was created with known concentrations of PB as described elsewhere [24,31].

### 2.8. Stress Response Assays

For the stress sensitivity assay, 5 × 10^4^ conidia from NK17 and the mutant strains were incubated on PLA plates and separately supplemented with 0.04% Congo red, 1 M KCl, and 2 M sorbitol for seven days at 28 °C. Each test was set in triplicate.

## 3. Results

### 3.1. Identification of the VeA, VelB, and LaeA Orthologs in Pestalotiopsis microspora

**Figure 1:**
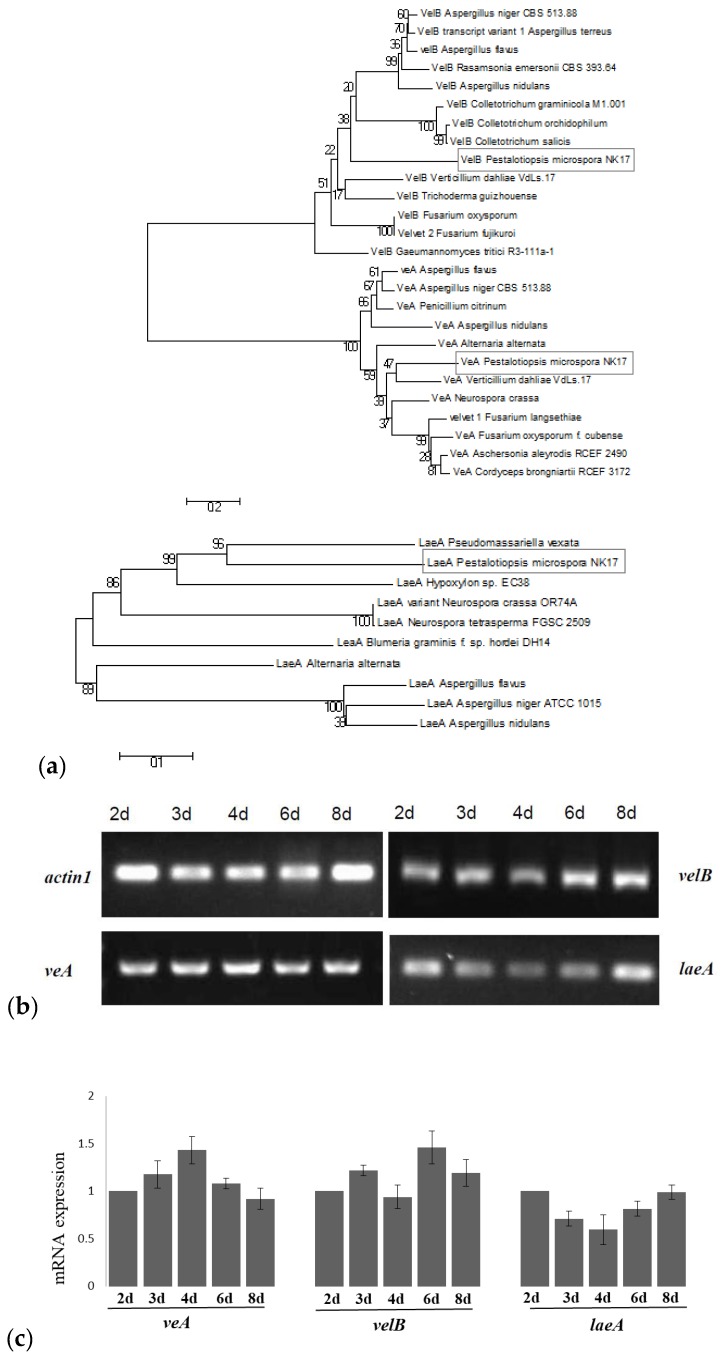
(**a**) Phylogenetic tree of fungal VeA, VelB, and LaeA proteins built by molecular evolutionary genetics analysis (MEGA) 5.1 software. Indicated by the grey rectangles are VeA, VelB and LaeA from *Pestalotiopsis microspora*. Phylogenetic relationships were inferred using the Neighbor-Joining method and bootstrap tested (1000 replicates). Branch lengths of the tree are drawn to scale and bootstrap support indicating at the branch sites. (**b**) Reverse transcription polymerase chain reaction (RT-PCR) for the mRNA of *veA*, *velB*, and *laeA* to show their expression. The top left panel was the messenger RNA (mRNA) of the actin-encoding gene *act1* used as the control in this study. (**c**) Expression levels by RT-PCR were estimated by densitometry and normalized to actin levels. Three experiments were done in each case. Error bars indicate standard errors.

Based on the genome sequence of NK17 (sequenced by BGI, Shenzhen, China; http://www.genomics.cn/index), two velvet-domain-containing proteins and a putative LaeA protein (GME14108_g, GME13740_g, GME5440_g) were identified in *P. microspora* using the protein basic local alignment search tool BLASTP algorithm (https://blast.ncbi.nlm.nih.gov/). They shared a 62% (*E*-value 2 × 10^−75^), 49% (*E*-value 3 × 10^−84^), and 47% (*E*-value 1 × 10^−89^) sequence identity with the velvet A protein from *Neurospora crassa* [32], velvet B protein from *A. terreus* [33], and LaeA protein from *Alternaria alternata* [34], respectively. Phylogenetic analysis by molecular evolutionary genetics analysis (MEGA) 5.1 software [35] suggested that these proteins respectively clustered with VeA, VelB, and LaeA proteins in other filamentous fungi, supporting the hypothesis that they are VeA, VelB, and LaeA proteins (Figure 1a).

Analysis for conserved structural domains revealed that the VeA (212 amino acids (aa)) protein contained one velvet domain (Figure S1) and the *velB* (377 aa) protein had two velvet domains (Figure S2). Similarly, in *A. nidulans*, VeA and VelB proteins contained one and two velvet domains, respectively. The LaeA (316 aa) protein in our fungus had an S-adenosylmethionine (SAM) binding site in the putative protein as in other LaeA proteins [10,15,18,36] (Figure S3). Amplification with reverse transcription PCR (RT-PCR) demonstrated that the three genes encoding VeA, VelB, and LaeA were constitutively expressed in NK17 (Figure 1b,c).

To investigate the function of *veA*, we replaced the genomic copy with a selection marker (the NK17 *ura3* gene) via homologous recombination (Figure S4). A portion of the *veA* ORF was replaced by the marker *ura3* in the recipient NK17-*△ura3* to obtain the deletion transformants. Three transformants were acquired by PCR screening as the candidates of *veA* deletion (Figure S5b). One of the candidates was picked for Southern blotting (Figure S5c). The complemented strain *△veA*-C was constructed by a procedure described in the Materials and Methods section (Figures S6 and S7) and verified by PCR amplification (Figure S5b) and by Southern blotting (Figure S5c). The transformant confirmed by both PCR amplification and Southern blotting was designated as *△veA*. The deletion and complemented strains of *velB* and *laeA* were confirmed by a similar procedure (Figure S8).

### 3.2. Involvement of VeA, VelB, and LaeA in Hyphal Growth and Conidiation

**Figure 2:**
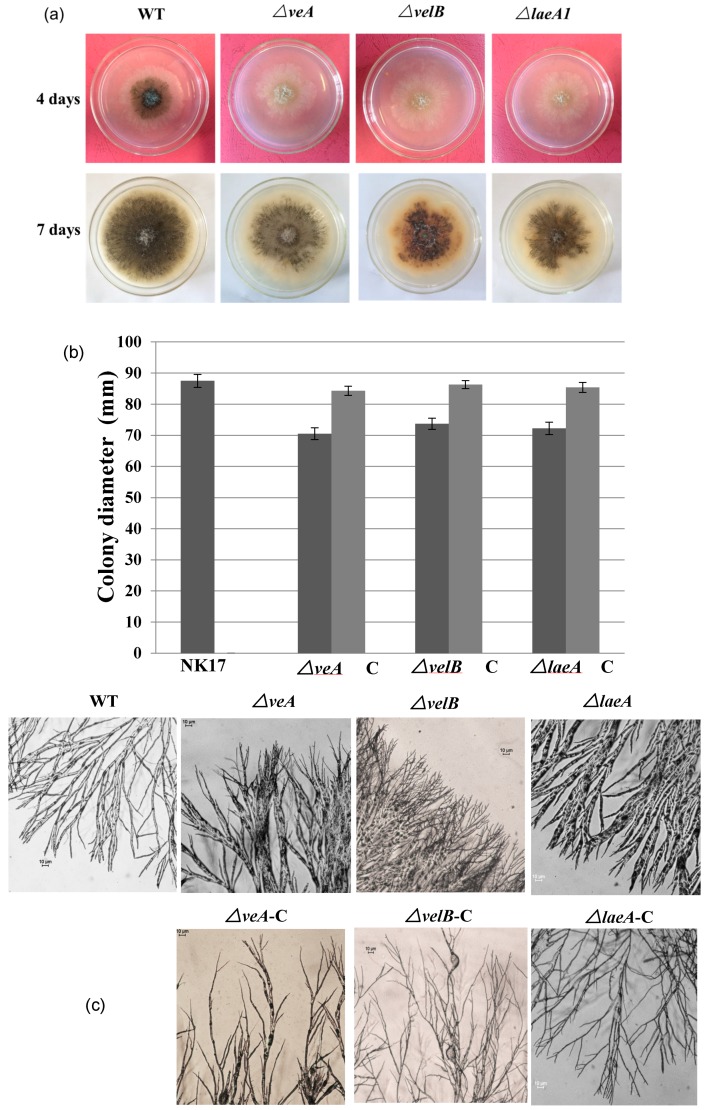

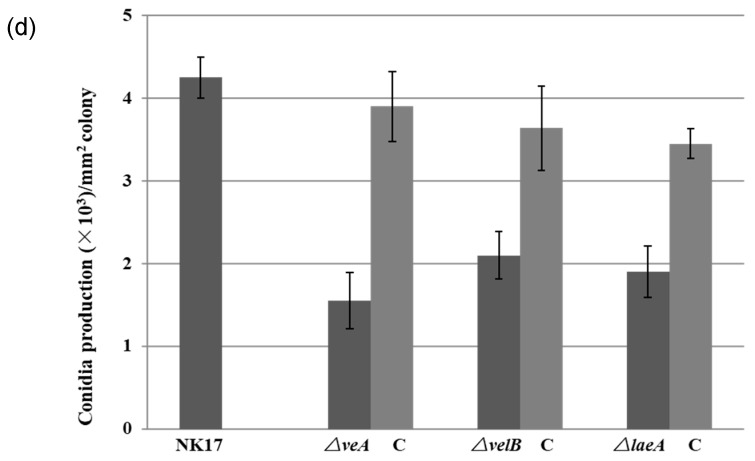
(**a**) Vegetative growth of the mutant strains *△veA*, *△velB*, and *△laeA* on potato lactose agar (PLA) plates grown for four days (top) and 7 days (bottom) at 28 °C under laboratory illumination. (**b**) Colony sizes of NK17, *△veA*, *△velB*, and *△laeA*, and the respective complement strains grown on PLA plates for seven days at 28 °C. Error bars represent standard deviation. (**c**) Microscopic analysis for hyphal tip branches of *△veA*, *△velB*, and *△laeA* grown on minimal medium (MM) plates for five days at 28 °C when compared with NK17 and the complemented strains. (**d**) Conidia production per mm^2^ of colony in NK17, *△veA*, *△velB*, and *△laeA,* and the respective complement strains were grown on PLA. Triplicate PLA plates for each strain were incubated at 28 °C for eight days. Error bars represent the standard deviation.

We performed several experiments to observe the phenotype changes in the deletion mutant strains. As shown in Figure 2a, the colony size of the mutants *△veA*, *△velB*, and *△laeA* grown on PLA after a 7-day incubation were distinguishably smaller than that of NK17. Assessment of the growth rate by colony diameters confirmed this observation (Figure 2b). Furthermore, microscopy suggested that the mutant strains exhibited morphological alternation. The hyphal diameter of *△veA* and *△laeA* appeared obviously thicker than that of NK17, i.e., 4.86 ± 1.84 μm and 6.25 ± 3.22 μm versus 1.52 ± 0.41 μm of the wild type NK17 (*p* < 0.01), respectively. Diameter data represent the means of 50 replicates. The *△velB* and *△laeA* strains formed denser and more branching hyphae at the periphery of colony on the MM plates. Complementation of the three mutants with corresponding wild-type copy restored the hyphal morphology (Figure 2c).

Furthermore, we could also see that the colony pigmentation and conidiation of the mutant strains were dramatically delayed (Figure 2a). We consequently conducted quantification to measure the number of conidia prepared from 8-day-old plates. The mutants *△veA*, *△velB*, and *△laeA* produced about 1.55 ± 0.34 × 10^3^, 2.1 ± 0.39 × 10^3^, 1.9 ± 0.31 × 10^3^ conidia/mm^2^, respectively, whereas NK17 produced about 4.5 ± 0.25 × 10^3^ conidia/mm^2^ (*p* < 0.01). The complemented strains *△veA*-C, *△velB*-C, and *△laeA*-C restored the conidiation to the numbers of 3.9 ± 0.42, 3.64 ± 0.51, 3.45 ± 0.18 conidia/mm^2^ (*p* < 0.01), respectively (Figure 2d). The complemented strains also restored the normal growth of the wild-type (WT) NK17 strain (Figure 2d). In addition, the deletion of the three genes delayed the conidiation by approximately one day. We did not observe differences on the hyphal growth and conidiation of the mutants grown in the dark and grown under light conditions. Taken together, the velvet components were required for conidiation and vegetative growth in *P. microspora* NK17.

### 3.3. Determination of Sensitivity of the Mutants to External Stresses

**Figure 3:**
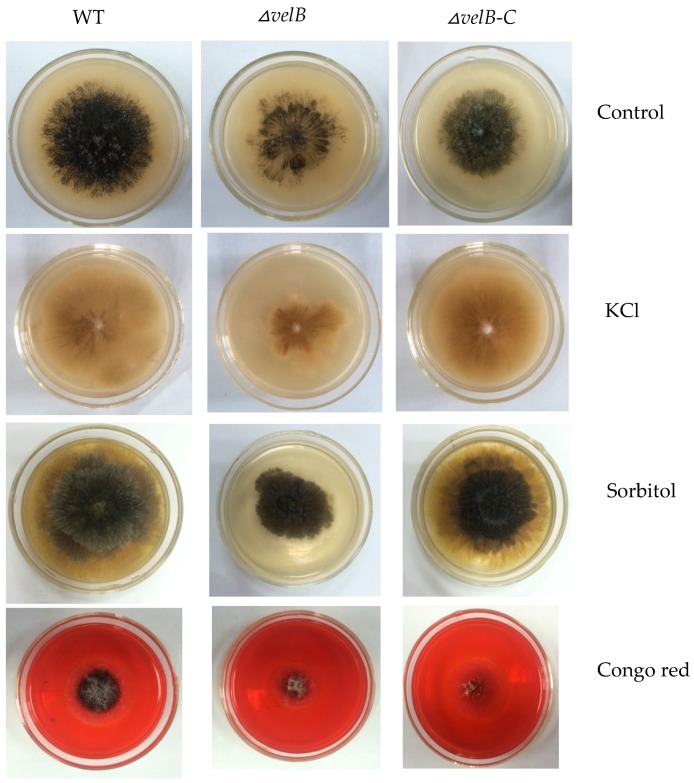
Sensitivity tests for NK17, *△velB*, and *△velB*-C strains. The strains incubated at 28 °C for five days on MM plates supplemented with 1 M KCl, 2 M sorbitol, or 0.04% Congo red. Each test was set in triplicate.

As loss of *veA* or *velB* in *Fusarium graminearum* increases resistance to osmotic stresses and cell wall damaging agent Congo red [37,38], we examined the tolerance in our mutants *△veA*, *△velB*, and *△laeA* to several stress conditions. In contrast, our study showed that the addition of 1 M KCl or 2 M sorbitol to PLA plates led to marked inhibition of growth of *△velB* through the size of the colony, while the condition showed little effect on the growth of the WT and the complementation strain (Figure 3). This result explicates that *velB* is required for cellular responses to osmotic changes.

The addition of 0.04% Congo Red to PLA resulted in significantly slower growth and less conidiation of *△velB* when compared with the controls (Figure 3), but little difference came to those of *△veA* (Figure S9) and *△laeA*. This result suggests that the deletion of *velB* affects the integrity of the cell wall in our fungus.

### 3.4. VeA, VelB, and LaeA Regulate Biosynthesis of Pestalotiollide B and Mycelial Pigments

In filamentous fungi, the production of secondary metabolites is coordinated by both pathway-specific and global transcriptional regulators [39,40]. The multicomponent global regulator velvet complex has been identified as a key regulator of secondary metabolite production in a number of fungi including *Aspergillus* and *Penicillium* spp.

**Figure 4:**
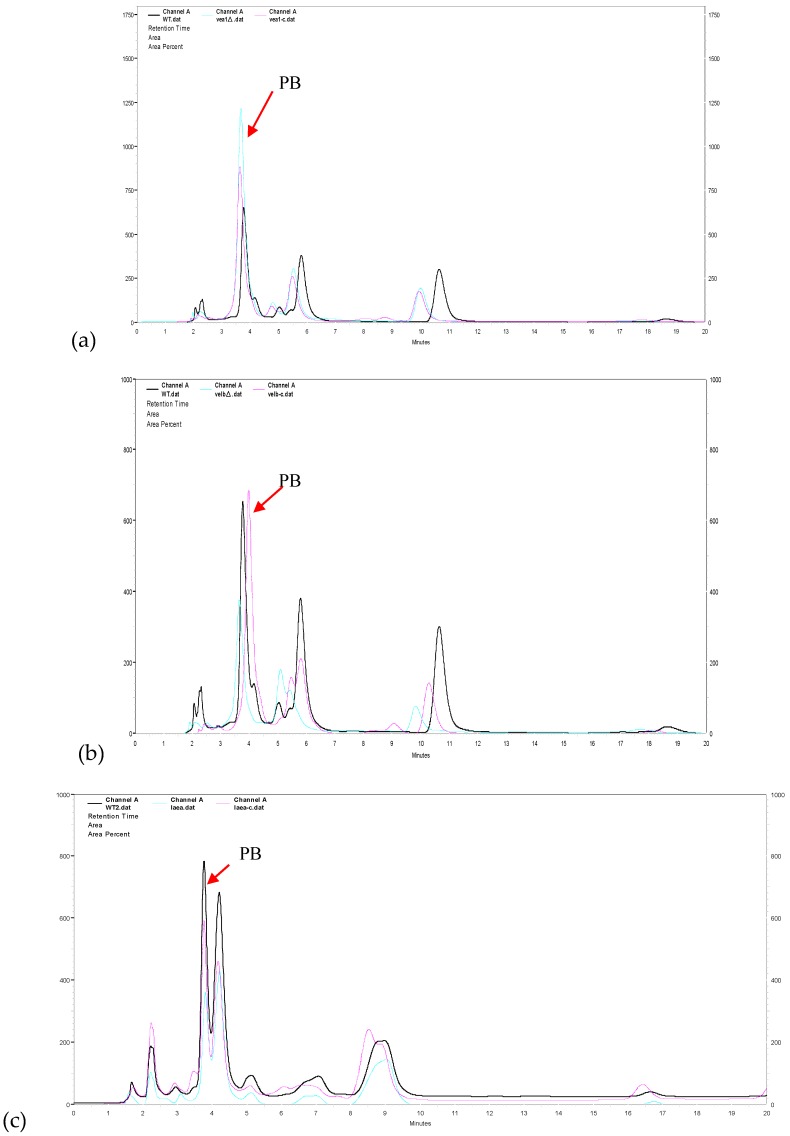

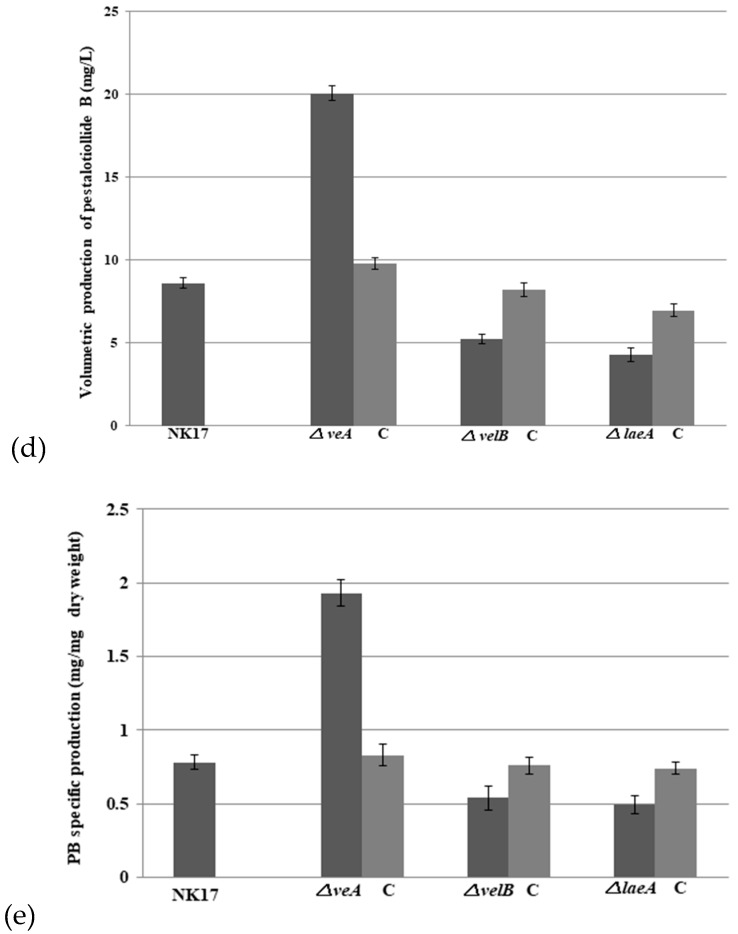
Effects of *veA*, *velB*, and *laeA* on secondary metabolism. High performance liquid chromatography (HPLC) profiling was conducted for secondary metabolites extracted from the liquid cultures of NK17 (black line), the three mutants (blue line), and the respective complement strains (pink line). Fungal strains were grown in 200 mL potato dextrose both, shaken at 28 °C, 180 rpm for seven days. The peak of pestalotiollide B (PB) is indicated by the red arrow. (**a**) HPLC profiling for *△veA* as shown in green. (**b**) HPLC profiling for *△velB* (green). (**c**) HPLC profiling for *△laeA* (in green). (**d**) Volumetric production of pestalotiollide B in NK17, *△veA*, *△velB*, and *△laeA* strains. (**e**) Qualification for the yield of PB in NK17, *△veA*, *△velB*, and *△laeA* strains by the dry weight of mycelium. HPLC profiling for each strain was performed in triplicate. Error bars indicate standard errors.

With the purpose to identify the role of the velvet complex components in the production of PB in NK17, we conducted HPLC profiling for the mutant strains *△veA*, *△velB*, and *△laeA*. The extracts were prepared with the protocol described in the Section 2. We found that *△velB* and *△laeA* mutants produced less PB relative to the WT NK17 (Figure 4a,c). On the other hand, PB production increased remarkably in *△veA* (Figure 4b). The yield of PB was quantified using a standard curve that was created with known concentrations of PB as described elsewhere [24]. The PB production in the *△velB* and *△laeA* strains were 5.24 ± 0.26 mg/L and 4.3 ± 0.41 mg/L, respectively. The complemented strains *△velB*-C and *△laeA*-C restored the yield of PB to nearly that of the wild-type level of PB (Figure 4d). The concentration of PB was 20.08 ± 0.44 mg/L in the liquid culture of *△veA*, whereas PB concentration for NK17 and the complement *△veA*-C were 8.61 ± 0.32 mg/L and 9.8 ± 0.35 mg/L, respectively. As seen earlier in this study, we showed that the deletion of the three genes led to a decrease in vegetative growth (Figure 2a,b), so we tested whether PB variation was caused only by the change of mycelial growth. The absolute yield of PB versus the dry weight (mg/mg) was calculated and is shown in Figure 4e. In a comparable consistence, deletion of *veA* led to a remarkable increase of PB production, suggesting an inhibitory role of *veA* in the biosynthesis of PB. In contrast, the quantitative results demonstrated that LaeA and VelB were required for the normal biosynthesis of PB.

**Figure 5:**
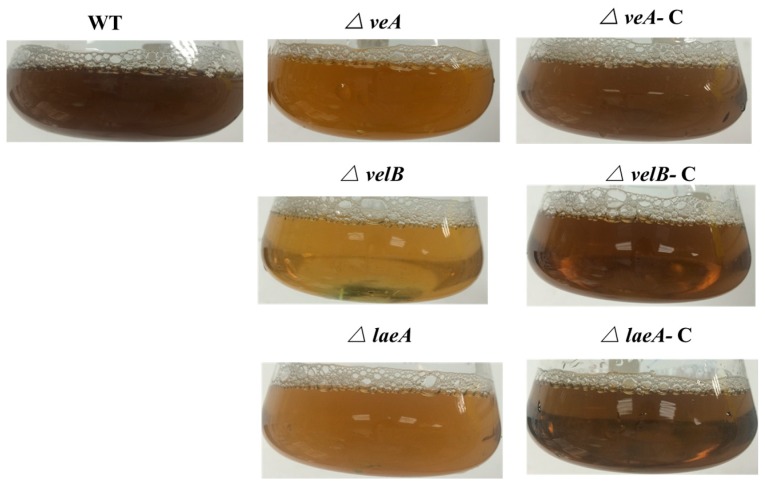
Impaired pigmentation in NK17, *△veA*, *△velB*, and *△laeA* strains. The strains were incubated in PLB at 28 °C for seven days. Complemented strains restored to the level of the wild type.

Through the color of the colonies on the 4-day plates (Figure 2a), we can see that the pigmentation of mycelium/conidia (since conidia are pigmented in this fungus) was severely reduced in the mutant strains, even after seven days of growth, all the mutants produced more pigment. This was obviously repeated in the liquid culture of the fungi (Figure 5). Thus, *veA*, *velB*, or *laeA* are critical for the biosynthesis of mycelial pigments in *P. microspora*.

**Figure 6:**
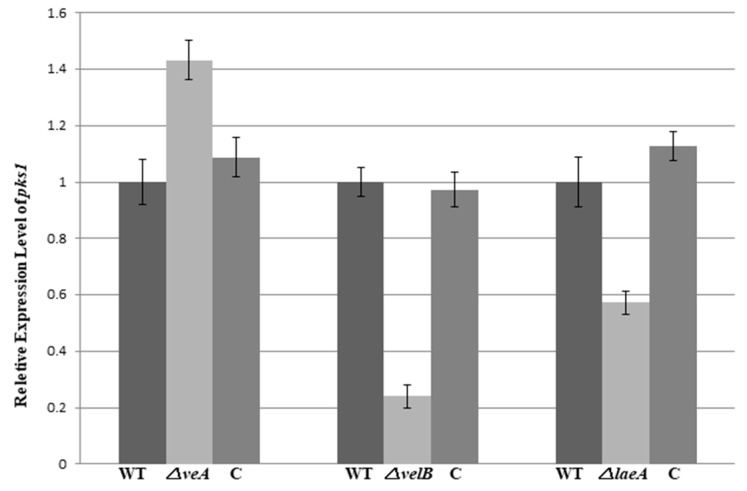
Quantitative analysis of *pks1* gene expression in NK17, *△veA, △velB*, and *△laeA* strains by quantitative real-time polymerase chain reaction (qRT-PCR). RNA from mycelium was collected on the 7-day of growth in PLB (PLA without agar) at 28 °C. Complemented strains were restored to the level of the wild type. The qRT-PCR was performed in triplicate, and error bars indicate the standard errors.

To further observe the roles of *veA*, *velB*, and *laeA* in the regulation of pigmentation, we carried out an assessment of *pks1* expression in the fungal strains. The gene *pks1*, a polyketide synthase encoding gene, is responsible for the pigment biosynthesis in conidia and mycelium in NK17 [28]. The qRT-PCR data showed that the mRNA level of *pks1* in the *△velB* and *△laeA* strain decreased by approximately 4.2-fold and 1.8-fold, respectively; however, in *△veA*, it increased by 1.4-fold (Figure 6). This result once again suggests that *veA*, *velB*, and *laeA* are global regulators in the biosynthesis of PB and pigments in *P. microspora*.

## 4. Discussion

Control of growth and development in filamentous fungi, often together with secondary metabolism has been at the heart of molecular mycology. A body of evidence suggests that secondary biosynthetic pathways of fungi are often co-regulated with asexual/sexual development in response to external cues including light [10,17,41]. For instance, *A. nidulans* produces asexual spores in light, but if under dark, it undergoes sexual reproduction preferentially [42,43] and increases the production of secondary metabolites [44]. Velvet proteins VeA and VelB, together with the global regulator LaeA have been well established as one of the regulatory complexes in a number of fungi in concerting the processes [17,41]. In *A. nidulans*, it has been shown that VeA is a bridge protein between secondary metabolism, fruit body formation, and light, whose expression increases during sexual development [11]. VeA transport into the nucleus is inhibited by the light [45]. It rather acts as a negative regulator of asexual development and antibiotic biosynthesis in *A. nidulans* and some other fungi [14,46,47,48,49]. However, distinct modes of action by the components of the complex exist in different fungi [41,47,49,50,51,52,53,54,55,56]. To illustrate the function of the conserved complex in *P*. *microspora*, we performed loss-of-function analyses on the genes *veA*, *velB*, and *laeA*, which are the equivalent components of the velvet complex. We found that the proteins were involved in plural biological processes in our fungus.

First, the loss of any one of *veA*, *velB*, and *laeA* in *P. microspora* caused a similar restricted vegetative growth in all three mutant strains (Figure 2), suggesting that they regulated vegetative growth, and even likely acted together. This observation, i.e., growth defects caused by mutation of the genes, has yet to be reported in other fungi. We also observed that the deletion of *veA* and *LaeA* led to hyphae morphological change in the diameter, but not the *velB* (Figure 2c), and implied intriguingly that VeA and LaeA work together in this process. This role of *veA* has also been found and reported previously in *Fusarium verticillioides* [50] and *Aspergillus chrysogenum* [51]. Our finding suggests a disparity in action of the two genes at least in our fungus, which was also later found in several phenotypes of the mutant strains. For instance, their effects on the expression of *pks1*, the polyketide synthase gene in the melanin biosynthetic pathway were differential. In this case, the deletion of *veA* resulted in a discernible elevation of the mRNA level of *pks1*, whereas it caused a dramatic fall in the level in *ΔvelB* and *ΔlaeA* (Figure 6). These results clearly suggested that *veA* and *velB* could function independently in different biological processes, with or without interacting with *laeA* in *P*. *microspora*.

One of the major functions of the velvet components is in the regulation of sexual, asexual reproduction and the secondary metabolite biosynthesis [10,17,19]. This has been reported in almost all the fungi investigated by far [10]. However, the roles appear variable in different fungi, suggesting an evolutionary adaptation in the proteins and LaeA to the environment. We demonstrated that the regulatory function was conserved in *P. microspora* in the development of the asexual stage and the production of secondary metabolites, i.e., PB. The action of velvet complex and LaeA in conidiation in our fungus included two different aspects, namely, the timing of conidiation and the capacity of conidia production. Conidiation was apparently delayed in all the three mutant strains (Figure 2a) and the number of conidia produced by them was also reduced to a level of less than half of the wild type (Figure 2d). The results reflected that on the one hand, VeA, VelB, and LaeA were likely to act together in this case, yet in a positive manner in the regulation of asexual development in *P. microspora*. Interestingly, the roles of the components have shown evolutionary divergence in asexual development. In *A. nidulans*, VeA, though a positive regulator in sexual development, inhibits asexual development [11]. This is the case in a number of other fungi, for instance, *N. crassa*, *F. graminearum*, *F. verticillioides*, *Cochliobolus heterostrophus*, and *Botrytis cinerea* [32,37,49,50]. Deletion of the *veA* gene in these fungi results in a significant increase in conidial production, demonstrating an inhibitory action of VeA. Nonetheless, in *Aspergillus carbonarius*, *Aspergillus parasiticus*, *F. fujikuroi*, *A. fumigatus*, *F. oxysporum*, and *Penicillium chrysogenum*, it has been described where VeA plays a similar role to *P. microspora* veA in asexual development [15,16,47,51,52,53]. As to the action of *velB* and *laeA*, at least in *A. nidulans*, *A. carbonarius*, and *P. chrysogenum*, their deletion led to impaired asexual sporulation, which was comparable to their counterparts in *P. microspora* [18,32,54,55].

As the initial aim of the work, we were interested in finding the possible regulatory role of the velvet complex in the production of secondary metabolites in *P. microspora*. A finding in this study was that the complex members VeA and velB/LaeA had opposite regulatory roles in the production of secondary metabolites, at least in the case of PB, a polyketide derivative, that is structurally related to CETP inhibitors dibenzodioxocinones [24,57,58]. Quantification and profiling by HPLC for PB revealed that VelB and LaeA were positively involved in the production of PB, since the loss of their genes caused a significant decrease of PB (Figure 4a–e). On the opposite side, the deletion of *veA* led to an increase of PB production, suggesting a negative role of VeA in PB production (Figure 4). This functional discrepancy of velvet proteins and LaeA in the secondary metabolism strikingly resembled their action in *pks1* transcription and in the conidiation stage as we mentioned earlier in this section (Figure 6) [28]. This outcome was comprehensible given the fact that, being a derivative of polyketide, melanin is, in a sense, a secondary product [31]. Once again, our results suggested that VeA, VelB, and LaeA could act independently in *P. microspora*. However, in other fungi, studies have suggested that *veA* was required for secondary metabolism [15,17,32]. In *A. nidulans*, VelB, VeA, and LaeA formed a protein complex to control sexual structure development and secondary metabolism [17]. Deletion of either VeA or VelB caused defects in secondary metabolite production. Taken together, the velvet family in different fungi seem to play divergent roles in the regulation of secondary metabolism.

Moreover, another novel role disclosed by this study was that the VelB protein was required for cell wall integrity (Figure 3). This was demonstrated by the sensitivity assay to the osmatic pressure (1 M KCl and 2 M Sorbitol) and the dye Congo red. Surprisingly, only the *△velB* strain exhibited hypersensitivity to osmotic stresses and to the dye Congo Red, but not the deletion mutants of *veA* and *laeA*. In yeast fungi, it has been well established that Congo red binds to the cell wall polysaccharides and sensitivity to the dye indicates a defect in the biosynthesis of the polymers [37,38]. Thus, our data supported the conclusion that VelB participates in the cell wall integrity of *P. microspora*. Sensitivity to osmotic stress can also be accounted by the cell wall defect in the mutant strain. At the molecular level, it has been clearly illustrated that, in baker’s yeast and some other fungi, responses to osmotic stress involved the high osmolarity glycerol (HOG) signal transduction pathway [59], therefore, it is intriguing to think that the HOG signaling is possibly blocked in the mutant *△velB*. The result is evidence of the view that VelB has crosstalk with the HOG signal transduction pathway in this fungus.

Last but not least, we previously reported that the G-protein-cyclic adenosine monophosphate (cAMP) signaling pathway in *P. microspora* NK17 was critical for the production of conidia and the biosynthesis of secondary metabolites [60]. Together with current results, this work raises the possibility that the G-protein signaling pathway, velvet complex, and LaeA interact with one or all the components during certain stages in the growth and development of this fungus.

## 5. Conclusions

We demonstrated in this study that the critical roles of the components of the velvet complex VeA and VelB, and LaeA were as regulators in the growth, conidial development, biosynthesis of secondary metabolites and cell wall integrity in *P. microspora*. Our data demonstrated that distinct roles of the components were required for different biological processes. Furthermore, the complex in *P. microspora* also showed functional divergence from the other fungal organisms. This work provides information for further understanding of the role of velvet complex in the biosynthesis of secondary metabolites such as the taxol and pestalotiollide B.

## Figures and Tables

**Table 1:** Primers used in this study. Under lines indicate the attB sequence.

Primer	Primer Sequence, 5′→3′
VeA-up-s	GGGGACAGCTTTCTTGTACAAAGTGGAAGGGTAGAACGGGACGACT
VeA-up-as	GGGGACTGCTTTTTTGTACAAACTTGTGCGGGAAGGGTAAGTGAG
VeA-down-s	GGGGACAACTTTGTATAGAAAAGTTGTTCCACCTATTGTTCGCTTGA
VeA-down-as	GGGGACAACTTTGTATAATAAAGTTGTGTCCTGGGCATCCTTGTT
Ura3(s)	GTCAAGACATCTGTTACCGTGG
Ura3(as)	GCAGGCGGGTAGTAGAGT
VeA(s)	GGGGACAGCTTTCTTGTACAAAGTGGAAGGGTAGAACGGGACGACT
VeA(as)	GGGGACAACTTTGTATAATAAAGTTGTCGACGGAAGCCTGTATCAA
Hyg(s)	CCGGTCGGCATCTACTCT
Hyg(s)	CGTTGCAAGACCTGCCTGAA
ART(F)	CATTCACAAGGCGGGAGA
ART(R)	CGAACAATAGGTGGAGGGTC
VelB-up-s	GGGGACAGCTTTCTTGTACAAAGTGGAAACGACGGTTGTGGTTCAG
VelB-up-as	GGGGACTGCTTTTTTGTACAAACTTGTAGGGCAATCCAGGTAAGC
VelB-down-s	GGGGACAACTTTGTATAGAAAAGTTGTTAGGTTATGATACAATCGGGTTA
VelB-down-as	GGGGACAACTTTGTATAATAAAGTTGTAGACAAGAGGTGCGGAAA
VelB(s)	CGCACAGCCCATTTAGAG
VelB(as)	CCAAGCCATCAGATCGTG
BRT(F)	GATGGAACCTGGACATACAA
BRT(R)	ACGGAGGAGGCGTGATAG
LaeA-up-s	GGGGACAGCTTTCTTGTACAAAGTGGAAAACCCTCCACCTCAACAACA
LaeA-up-As	GGGGACTGCTTTTTTGTACAAACTTGTTAGCAAGCCAATACACGATG
LaeA-down-s	GGGGACAACTTTGTATAGAAAAGTTGTTGGTTGCCCTTGAGCCTTGAA
LaeA-down-as	GGGGACAACTTTGTATAATAAAGTTGTCGCCGTATAAATGTACCTTGC
LaeA(s)	GGGGACAGCTTTCTTGTACAAAGTGGAATGCCAGGTTGTTCAGTAA
LaeA(as)	GGGGACAACTTTGTATAATAAAGTTGTGAGTCGGGCGGGTAGATT
LRT(F)	ACCCTCCACCTCAACAAC
LRT(F)	CATTAGCAAGCCAATACAC
Actin1(s)	GTCGCTGCCCTCGTTATC
Actin1(as)	CGAGAATGGAACCACCGAT

## Supplementary Materials

The following are available online at http://www.mdpi.com/2073-4425/9/3/164/s1, Figure S1:VeA information, Figure S2: VelB information, Figure S3: LeaA information, Figure S4: Construction of *veA* deletion and complement strains, Figure S5: Deletion of *veA* in NK17, Figure S6: Schematic of pOSCAR4.1 and the vector pOSCAR-VeA-C for gene complementation was constructed by BP clonase, Figure S7: Schematic of the complement vector pOSCAR-VeA-C and construction of complemented strain *△veA*-C. Figure S8: (a) Southern blot analysis of the *△velB* mutant. (b) PCR verification for *△laeA* mutants (top) and *△laeA*-C strains (bottom), Figure S9: Sensitivity tests for NK17 and *△velA* strains.
