# Supplementary material for: Distinct Roles of Velvet Complex in the Development, Stress Tolerance, and Secondary Metabolism in Pestalotiopsis microspora, a Taxol Producer
Source: Genes (Basel). 2018 Mar 14;9(3):164. doi: 10.3390/genes9030164 (PMC5867885; doi:10.3390/genes9030164)
Supplement: Supplementary file 1 [file genes-09-00164-s001.pdf]

SUPPLEMENTARY MATERIAL:

(a)

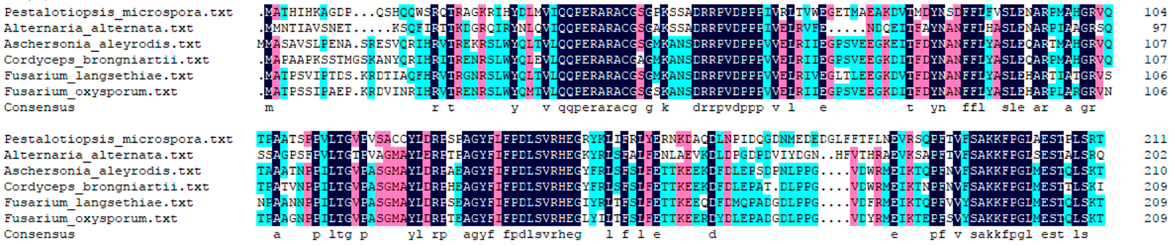

(b)

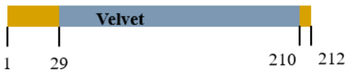

Figure S1. VeA information**. (a)** Alignment of the deduced amino acid sequence of VeA in *P. microspora* and the VeA orthologs in other fungi. The amino acid sequences were aligned using DNAMAN (Version 8.0) software. **(b)** Schematic of the structure of *veA* encoding protein from *Pestalotiopsi*s *microspore* analyzed by protein basic local alignment search tool (BLASTP) of NCBI (https://blast.ncbi.nlm.nih.gov).

(a)

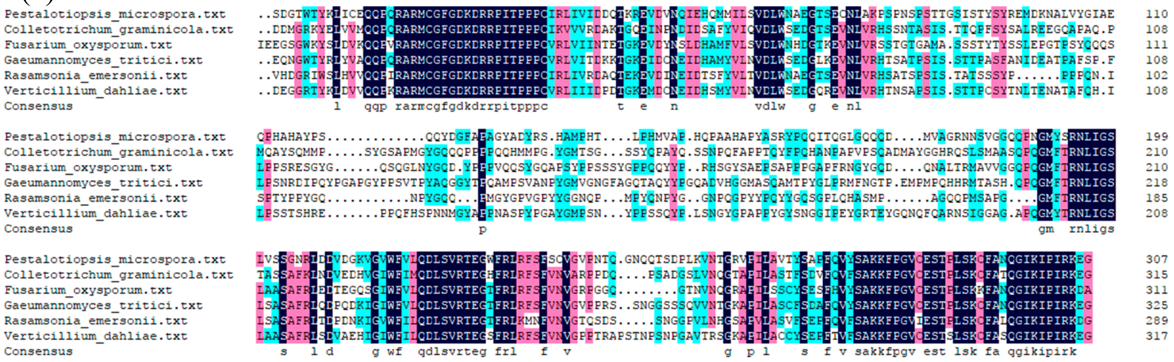

(b)

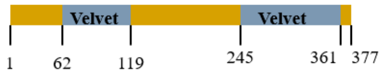

Figure S2. VelB information. **(a)** Alignment of the deduced amino acid sequence of VelB in *P. microspora* and the VelB orthologs in other fungi. The amino acid sequences were aligned using DNAMAN (Version 8.0) software. **(b)** Schematic of the structure of *velB* encoding protein from *P. microspore* analyzed by BLASTP of NCBI.

(a)

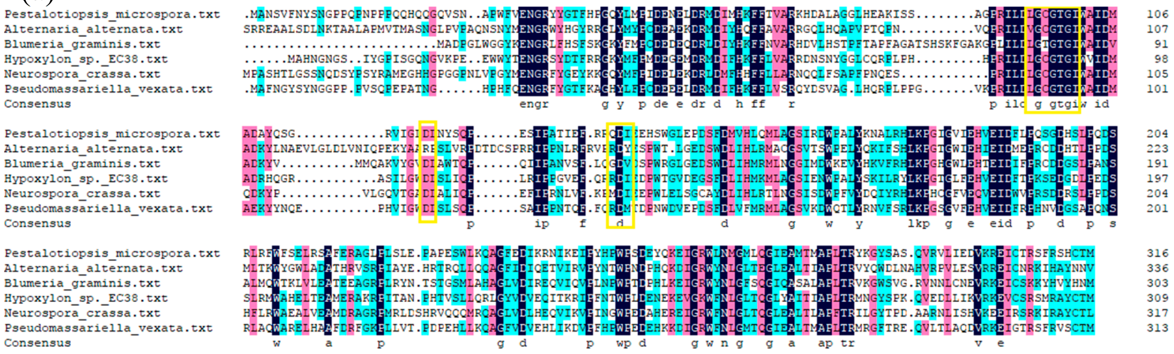

(b)

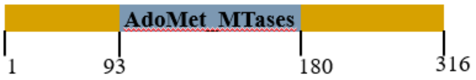

Figure S3 LeaA information. **(a)** Alignment of the deduced amino acid sequence of LaeA in *P. microspora* and the LaeA orthologs in other fungi. The amino acid sequences were aligned using DNAMAN (Version 8.0) software. **(b)** Schematic of the structure of *laeA* encoding protein from *P. microspore* analyzed by BlastP of NCBI. Conserved protein methyltransferase S-adenosylmethionine binding sites are boxed.

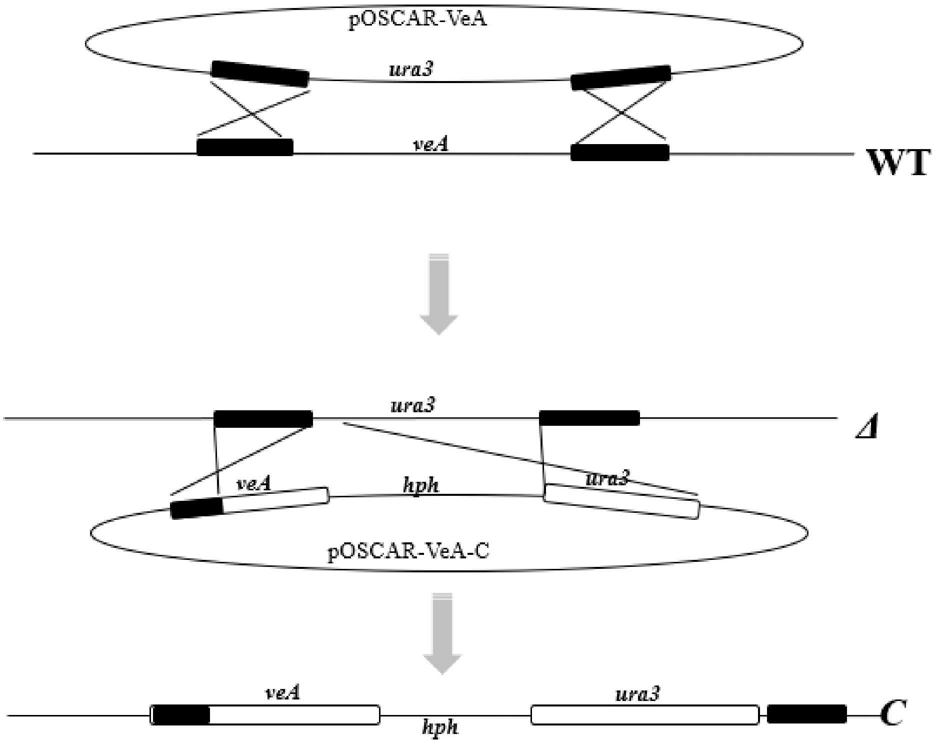

Figure S4. Construction of *veA* deletion and complement strains. Diagrams showing the strategy for generating *veA* deletion and complement strains by homologous recombination.

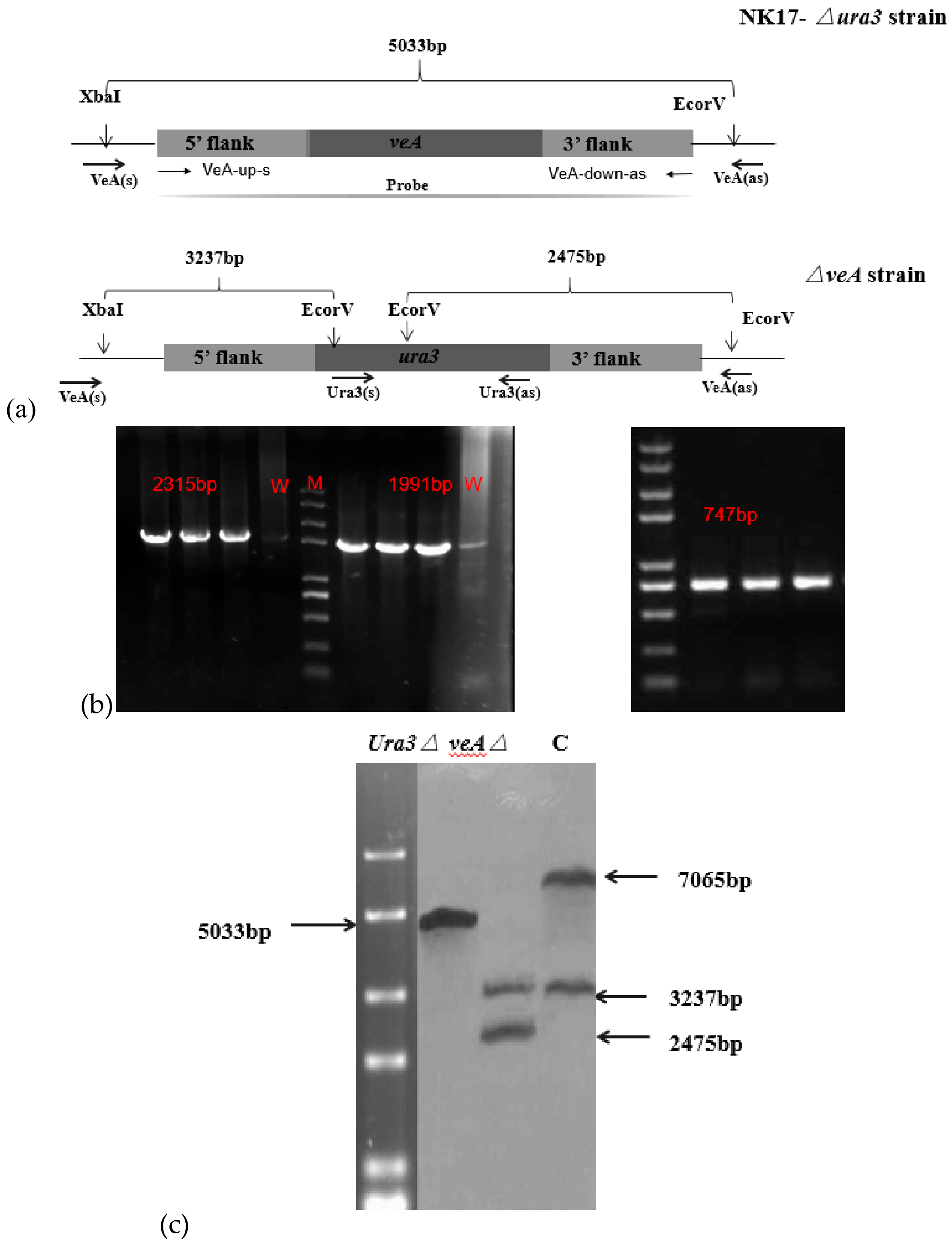

Figure S5. Deletion of *veA* in NK17. **(a)** Schematic of *veA* allele in NK17-*ura3*△ and *ura3* in *veA*△. The primers used for PCR confirmation of *veA*△, the location of the recognition sites of Xba I, Ecor V, and the probes used for Southern blot were shown. **(b)** Screening of *veA*△ (top) *veA*△ -C transformants by PCR. The expected size of PCR products with primer pairs VeA(s)/Ura3(as) and VeA(as)/Ura3(s) were 2315 bp (left) and 1991 bp (right) for *veA*△, respectively. A pair of primer Hyg(s) and Hyg(as) generated 747 bp sequence in *veA*△ -C. M is molecular marker, W presents NK17 strain (Trans2K plusII, TransGen, China). **(c)** Southern blot analysis of the *veA*△ mutant. Genomic DNA from NK17-*ura3*△, *veA*△ or *veA*△-C was digested with Xba I and Ecor V, and probed with a 1.7-kb *veA* fragment. Digestion of the NK17-*ura3*△ genome yielded a 5.0 kb band, whereas the *veA*△ had two bands, 3.2 and 2.5kb in length. Two bands, 3.2 and 7.0 kb were detected for complemented strain *veA*△-C (Schematic of construction of the complemented strain was represented in Figure S4).

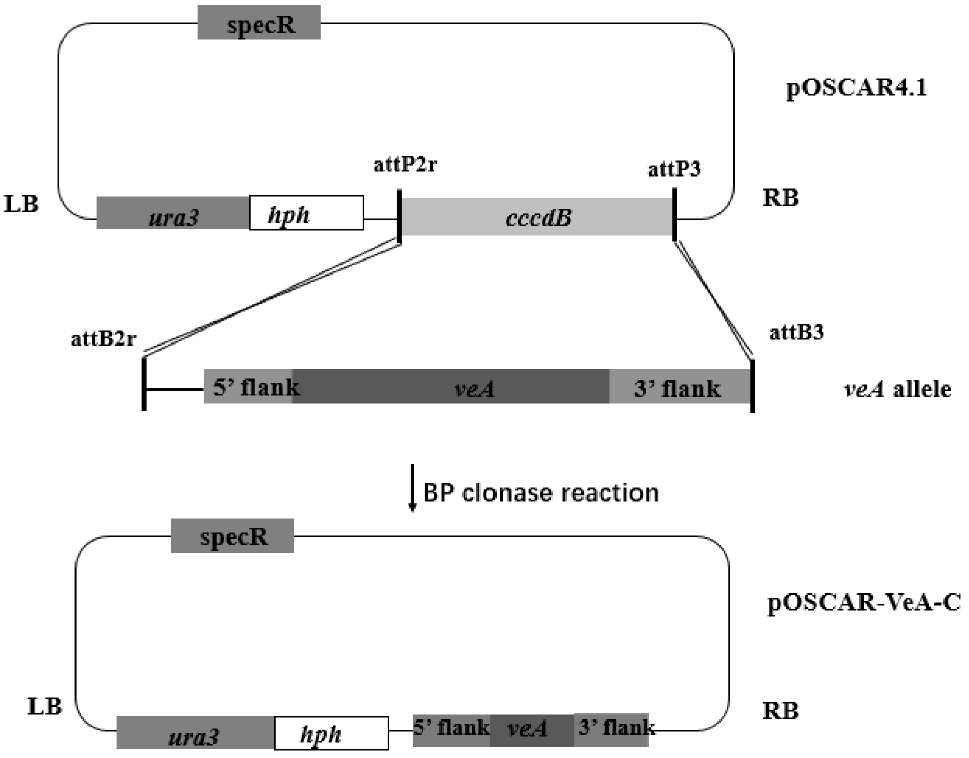

Figure S6. Schematic of pOSCAR4.1 and the vector pOSCAR-VeA-C for gene complementation was constructed by BP clonase. Location of primers used to amplify the *veA* allele were indicated.

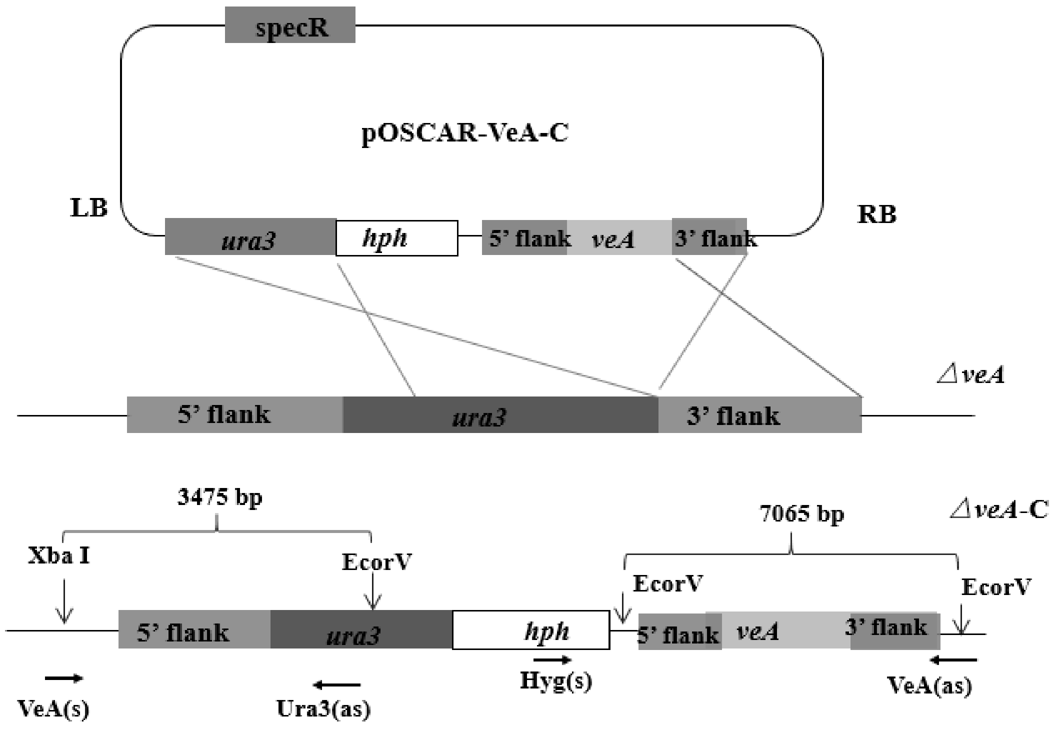

Figure S7. Schematic of the complement vector pOSCAR-VeA-C and construction of complemented strain △*veA*-C. The location of the recognition sites of of XbaI and EcorV are indicated.

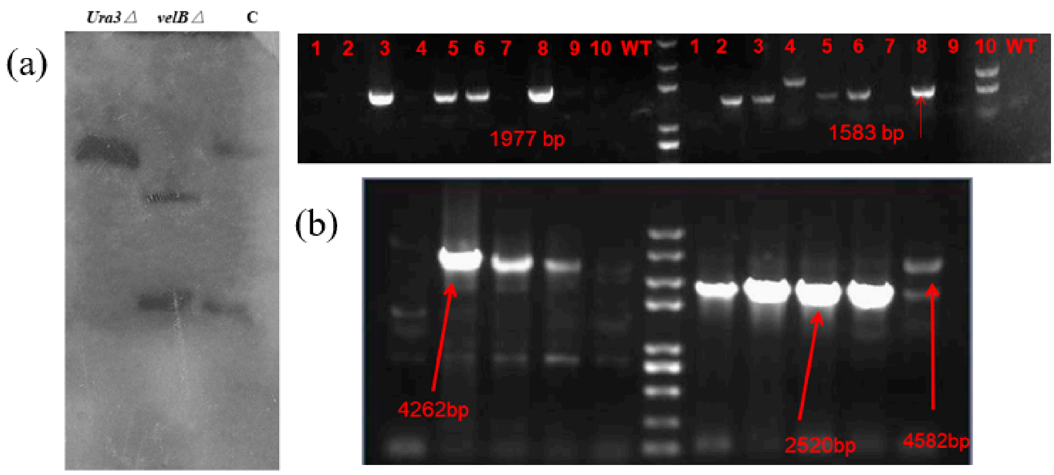

Figure S8. (a) Southern blot analysis of the △*velB* mutant. Genomic DNA from NK17- △*ura3*, △*velB* or △*velB*-C was digested with Sac probed with a 3.3-kb *velB* fragment. Digestion of the NK17- △*ura3* genome yielded a 6.6 kb band, whereas the △*velB* had two bands, 4.5 and 2.3kb in length. Two bands, 7.7 and 2.3 kb were detected for complemented strain △*velB*-C. **(b)** PCR verification for △*laeA* mutants (top) and △*laeA*-C strains (bottom). The expected size of PCR products with primer pairs VelB(s)/Ura3(as) and VelB(as)/Ura3(s) were 2315 bp (left) and 1991 bp (right) for △*velB*, respectively. The anticipated length of PCR product with primer pair LaeA-up-s/Hyg(R) was 4262 bp for the complemented strain △*laeA*-C, while no band was seen for △*laeA*; The predicted PCR product with primer pair LaeA-down-as/ura3(s) was 2520 bp in length for complemented strain, while 4582 bp for △*laeA*.

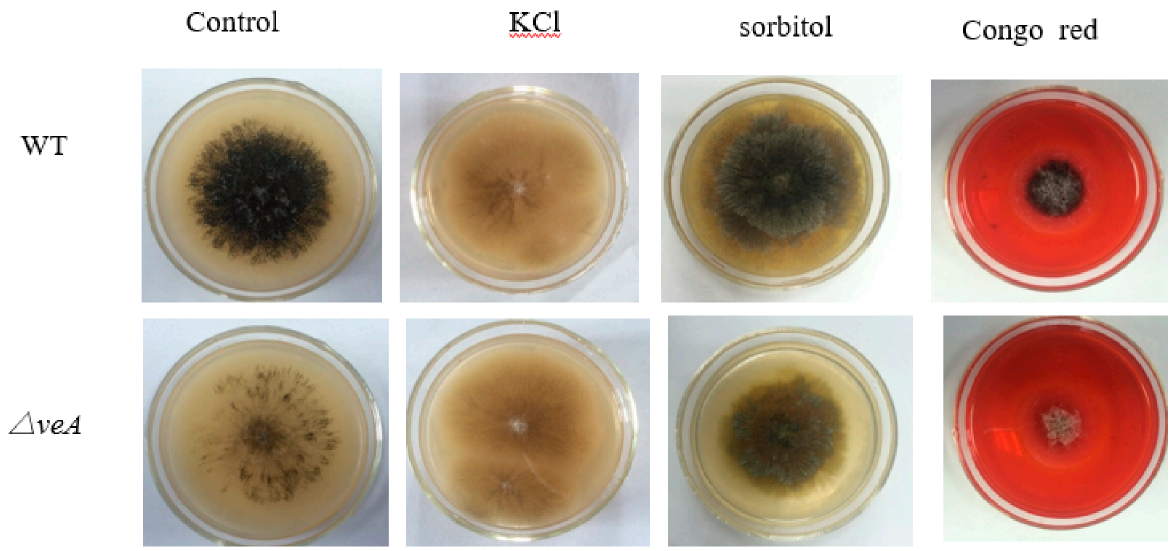

Figure S9. Sensitivity tests for NK17 and △*velA* strains. The strains incubated at 28 °C for five days on MM plates supplemented with 1M KCl, 2 M sorbitol, or 0.04% Congo red.
